# Moveable Kidney

**Published:** 1897-12-04

**Authors:** 


### MOVEABLE KIDNEY.

It is not so very long ago that the question was seriously asked whether, in fact, there was such a thing as a floating kidney, and perhaps even now there are practitioners who would hesitate to admit that the condition known as moveable kidney is at all common. If we look for moveable kidney as an isolated condition —a thing occurring separately and by itself—perhaps they are right; but if we regard it as but part of that general condition of laxity, which, in its varying degrees, constitutes what is known as enteroptosis, a more or less pendulous condition of all the viscera, then it is much more frequently met with. This is a matter of some importance, especially in regard to the question of nephrorrhaphy, for while the results of this operation are admittedly good in many cases, it does become a question whether it is wise to subject a patient to operation of a kidney, the mobility of which is but a part of a general relaxation and down-falling of all the viscera.

The symptoms of nephroptosis are now sufficiently well recognised; the shifting tumour, the weight and dragging, the occasional colicky pains, the sickening feeling when the displaced organ is pressed upon, together with the disturbances of the digestive organs and those more violent paroxysms which arise from obstruction of the ureter or renal vein, are well-known signs pointing to nephroptosis. It must be remembered, however, that in many cases in which the kidney is moveable symptoms are absent; and again, that even in those cases in which the suffering may be great it may not be continuous, varying much with the condition of the patient's health.

At the last meeting of the Medical Society Dr. Symons Eccles related a series of cases which bore upon this point, and showed that by massage and exercises directed to the development of the abdominal muscles, by what is termed "rest cure," the tendency of which is to replace the fat around the kidney, the loss of which has often been observed as one of the conditions associated with its mobility, and by the application of an appropriate pad and belt, it is sometimes possible to retain the kidney in its proper place, to give great relief to the symptoms, and in some cases to produce a cure quite comparable to that which is obtained by the operation of nephrorrhaphy. In view of these results he thought that sufferers from floating kidneys should be first treated by rest, massage, and exercises before they were exposed to the risks of operation, risks which certainly existed, however small they might be rendered by the skill of the operator.

We would by no means throw cold water on the treatment of nephroptosis by operation for fixing the kidney; but the association of this condition in some cases with a more or less general enteroptosis clearly removes such cases into a different category from those of ordinary "moveable kidneys," and, so far as general enteroptosis in its less severe degrees is capable of treatment by the means described by Dr. Eccles, the kidney condition will be relieved along with the rest. The observations detailed in regard to the kidney and the function of the perinephric fat only throw into all the stronger light the importance of making a careful physical investigation in all cases of abdominal disorder. Glenard's disease must always be borne in mind. A prominent belly is not always due to either tumour, fat, or dropsy; and if examined in the upright posture many a case of neurasthenic dyspepsia will be found to be due to a down dragging of the abdominal contents.

